# 112 A Virtual Vaccine Clinic Augments Adult Vaccination Rates in Primary Care Clinics

**DOI:** 10.1017/ash.2026.10529

**Published:** 2026-06-23

**Authors:** Karina Madureira Ramalho, Jessika Valeska Martins Ramos Alcara, Ariane Nascimento, Elba Gonves

**Affiliations:** 1 SHEA; 2 Hospital Unimed Sergipe; 3 Unimed SE

## Abstract

**Background:** Antimicrobial resistance (AMR) is a major global threat to patient safety and healthcare systems, largely driven by inappropriate antimicrobial use. Antimicrobial Stewardship Programs (ASPs) are recommended to optimize prescribing practices and mitigate resistance; however, data on their impact in private hospitals, particularly in middle-income countries, remain limited. The objective of this study is to describe the implementation of an Antimicrobial Stewardship Program and evaluate its clinical, microbiological, and economic impact in a private hospital. **Methods:** We conducted a retrospective observational before–after study in a private hospital, comparing a pre-intervention period (January–December 2022) with a post-intervention period (January 2023–December 2025). The ASP was implemented by a multidisciplinary team and included development of institutional antimicrobial treatment and surgical prophylaxis guidelines, prospective audit with feedback, restriction and pre-authorization of selected broad-spectrum antimicrobials, and integration among clinical pharmacy, infectious diseases, microbiology, and infection prevention services. Outcomes included antimicrobial prescribing conformity, antimicrobial consumption, incidence of multidrug-resistant organisms (MDROs), and direct antimicrobial acquisition costs. **Results:** Antimicrobial prescribing conformity increased from 86% in the pre-intervention period to consistently above 95% during the post-intervention years. Analysis of 720 clinical cultures identified 62 MDRO cases (8.7%) in the baseline period. Following ASP implementation, gram-positive multidrug-resistant organisms declined progressively, with no cases detected after 2024, while resistance among gram-negative organisms showed variable trends over time. An initial reduction in the use of broad-spectrum antimicrobials, particularly carbapenems, was observed after program implementation. Annual antimicrobial costs decreased from R$ 842,903.75 (≈ US$ 155,797) in 2022 to R$ 568,570.77 (≈ US$ 105,210) in 2023, followed by R$ 842,903.75 (≈ US$ 155,797) in 2022 to R$ 568,570.77 (≈ US$ 105,210) in 2023, followed by increases in 2024 – R$ 996,912.08 (≈ US$ 184,445) and 2025 – R$ 1,070,034.74 (≈ US$ 197,856), coinciding with a substantial increase in the number of patients receiving antimicrobials (from 9,704 in 2022 to 15,018 in 2025).increases in 2024 – R$ 996,912.08 (≈ US$ 184,445) and 2025 – R$ 1,070,034.74 (≈ US$ 197,856), coinciding with a substantial increase in the number of patients receiving antimicrobials (from 9,704 in 2022 to 15,018 in 2025). **Conclusions:** Implementation of a structured ASP in a private hospital was associated with improved prescribing quality, reduced initial use of broad-spectrum antimicrobials, favorable trends in gram-positive resistance, and mitigation of cost escalation despite increased patient volume. These findings highlight antimicrobial stewardship as a scalable and effective strategy to improve patient safety and contain antimicrobial resistance in private healthcare settings.

